# Hazardous, harmful, and dependent alcohol use in healthcare professionals: a systematic review and meta-analysis

**DOI:** 10.3389/fpubh.2023.1304468

**Published:** 2023-11-28

**Authors:** Lauren Halsall, Patricia Irizar, Sam Burton, Sara Waring, Susan Giles, Laura Goodwin, Andrew Jones

**Affiliations:** ^1^Spectrum Centre for Mental Health Research, Division of Health Research, Lancaster University, Lancaster, United Kingdom; ^2^Department of Sociology, School of Social Sciences, Faculty of Humanities, University of Manchester, Manchester, United Kingdom; ^3^Department of Women and Children’s Health, School of Life Course and Population Sciences, King’s College London, London, United Kingdom; ^4^School of Psychology, Faculty of Health, Liverpool John Moores University, Liverpool, United Kingdom; ^5^Department of Psychology, Institute of Population Health, University of Liverpool, Liverpool, United Kingdom

**Keywords:** meta-analysis, alcohol, drinking, health personnel, occupational health, COVID-19

## Abstract

**Background:**

Healthcare professionals work in high-pressured and demanding environments, which has been linked to the use of alcohol as a coping strategy. This international review aimed (i) to determine the pooled prevalence of hazardous, harmful, dependent, and frequent binge drinking in healthcare professionals, and (ii) to explore factors associated with variation in these outcomes.

**Methods:**

Scopus, MEDLINE, and PsycINFO were searched from 2003 to 17th November 2022, for studies reporting a prevalence estimate for any outcome among healthcare professionals. Random-effects meta-analyses determined pooled prevalence estimates. Sub-group analyses were conducted, stratifying the meta-analyses by pandemic period *vs* pre-pandemic period. Meta-regressions explored factors that were associated with variation in the outcomes. PROSPERO (CRD42020173119).

**Results:**

After screening 9,108 records, 64 studies were identified as eligible. The pooled prevalence was 19.98% [95% Confidence Intervals [CI]: 16.05–24.23%] for hazardous alcohol use (*K* = 52), 3.17% [95% CI: 0.95–6.58%] for harmful drinking (*K* = 8), 14.59% [95% CI: 7.16–25.05%] for dependent drinking (*K* = 7), and 17.71% [95% CI: 8.34–29.63%] for frequent binge drinking (*K* = 11). The prevalence of hazardous drinking was greater during the pandemic (28.19%) compared with pre-pandemic estimates (17.95%), though this was not statistically significant (*p* = 0.049). Studies including all hospital staff (32.04%) showed higher prevalence estimates for hazardous drinking compared with studies of doctors (16.78%) and nurses (27.02%).

**Conclusion:**

Approximately one fifth of healthcare professionals drink to hazardous levels, with higher prevalence estimates observed during the COVID-19 pandemic. It may be that healthcare professionals used alcohol to cope with the additional trauma and stressors. Further research is needed to investigate whether this is sustained in the post-pandemic period.

## 1 Introduction

Research shows that healthcare professionals experience occupational strains (1), including frequent exposure to trauma, and emotionally demanding and interpersonal stressors (2). These stressors have been linked to burnout, poor mental health, and maladaptive coping strategies such as using alcohol to cope (3, 4). Despite this, United Kingdom and international evidence indicates similar, or sometimes slightly lower, prevalence estimates of hazardous (drinking patterns associated with an increased risk of adverse health events) or harmful alcohol use (drinking patterns associated with known alcohol harms) in healthcare professionals compared to the general population (5–11). In addition, a recent meta-analysis estimated the prevalence of hazardous alcohol use in health professionals to be 13%, which was lower than prevalence estimates for other trauma-exposed occupations, e.g., police officers (12). However, most of the available studies are limited due to small sample sizes. The lower prevalence estimates among healthcare workers may also reflect confidentiality concerns or fears of disciplinary action following disclosure of hazardous or harmful alcohol use (9). Concerningly, harmful drinking (defined as >2 standard drinks per day) in healthcare professionals has also been shown to increase with years in service and hours worked (11).

The pressures and demands faced by healthcare professionals have been exacerbated during the recent COVID-19 pandemic, with global evidence demonstrating the detrimental impact on mental health, burnout and suicidal ideation among healthcare professionals (13–16). After the 2003 SARS outbreak, healthcare professionals reported increases in health risk behaviors, such as alcohol use and smoking (17). Emerging evidence in relation to COVID-19 highlights similar trends for alcohol use in healthcare professionals (18–20). Based on previous pandemics, these adverse outcomes could last for more than three years post-pandemic recovery (21). Ensuring a healthy workforce is crucial for staff, organizations, and wider society, as alcohol use is positively associated with sickness absence (22), which could pose subsequent adverse consequences for waiting times and patient safety. Examining the impact of COVID-19 on alcohol use on healthcare professionals is important for identifying the scale of the issue, informing policy decisions regarding investment in support services, and long-term service planning to promote a healthy workforce by preventing and reducing alcohol-related harms among healthcare workers.

To date, only one study has comprehensively reviewed the level of hazardous, harmful, and dependent alcohol use (characterized by tolerance, uncontrollable drinking, and physiological dependence which can result in withdrawals) or binge drinking (characterized by heavy drinking in a short space of time), across trauma-exposed occupations, which included healthcare professionals (12). This included healthcare professionals but did not consider the impact of COVID-19. The association between alcohol use, burnout and poor mental health in healthcare professionals have also yet to be comprehensively reviewed. Accordingly, the current systematic review seeks to explore the prevalence of hazardous, harmful, and dependent alcohol use, and frequent binge drinking in healthcare professionals, both before and during the COVID-19 pandemic. The protocol for this review is pre-registered with PROSPERO (CRD42020173119). This review aims to address the following research questions:

1. What is the prevalence of hazardous, harmful, dependent, and binge drinking, in healthcare professionals?
2. Does the prevalence of these outcomes differ among studies conducted during the COVID-19 pandemic (i.e., from March 2020) compared to studies that were conducted before the pandemic (i.e., before March 2020)?
3. Are there variations in the outcomes depending on the level of burnout or poor mental health within study samples?
4. Are there variations in the outcomes depending on socio-demographic factors of study samples (age, gender), or study variables (study quality, response rate)?

## 2 Materials and methods

### 2.1 Eligibility criteria

The “CoCoPop” mnemonic for reviews assessing prevalence and incidence data was used to determine inclusion and exclusion criteria (23). CoCoPop comprises of condition (i.e., health condition, disease, symptom, event, or factor), context (i.e., the environmental factors that impact on the prevalence or incidence of the condition) and population (i.e., population characteristics).

#### 2.1.1 Condition

The primary outcome of interest was alcohol use. This included any prevalence estimate for hazardous, harmful or dependent alcohol use, using a standardized measure, such as the 10-item Alcohol Use Disorder Identification Toolkit (AUDIT) (24, 25) or 3-item AUDIT-Consumption (AUDIT-C) (26), Timeline Follow Back (27) or the CAGE (Cut, Annoyed, Guilty, Eye-opener) questionnaire (28). We defined outcomes as hazardous, harmful, or dependent alcohol use, depending on the measures and criteria used in each study, which sometimes differed from the definitions used by authors [e.g., if a score of 4 or more on the AUDIT-C was defined as alcohol misuse, we would define it as hazardous alcohol use (26)]. Studies were also included if they reported a measure of frequent binge drinking (i.e., drinking heavily over a short space of time). The criteria used to define frequent binge drinking vary across countries and studies (e.g., 5 or more drinks on one occasion). Studies examining any substance use without specifying alcohol use were excluded.

The secondary outcomes of interest were standardized measures of poor mental health, e.g., depression, anxiety, or post-traumatic stress disorder (PTSD), and burnout. Burnout is usually measured using the validated Maslach Burnout Inventory (MBI) (29), which has previously been used to examine burnout in healthcare professionals (30–32). Any standardized measures of mental health were included (i.e., self-report screens and clinician administered assessments). Studies that only included a sub-population of participants with a physical or mental health condition were excluded. As this was a secondary outcome, we included studies that did not have a measure of poor mental health or burnout.

#### 2.1.2 Context

Geographical location data was used to determine differences in alcohol consumption across locations. As an additional aim, we sought to examine whether prevalence estimates for hazardous, harmful, dependent or binge drinking were different during COVID-19 (March 2020 to search date) compared with prior to the pandemic. We excluded studies which measured alcohol use after a major sentinel event, such as a hurricane.

#### 2.1.3 Population

The population of interest was healthcare professionals. This included doctors (i.e., surgeons, general practitioners, consultants, physicians, etc.) nurses, midwives, paramedics, dentists, pharmacists, and mental health practitioners. Medical students were excluded but doctors in residency, i.e., doctors in training for a given specialty (33), were included. Studies were included if subjects were of a working age (i.e., 16 years old) and retired samples were excluded.

### 2.2 Search strategy

To identify articles, we conducted a literature search using the databases: Scopus, MEDLINE and PsycINFO, from 2003 to 17th November 2022. Search terms describing healthcare professionals and alcohol use, outlined in the Supplementary materials, were used as free text terms and combined with Boolean operators. Peer-reviewed journal articles and grey literature (e.g., pre-prints, theses) written in English, were eligible for inclusion.

### 2.3 Data collection

#### 2.3.1 Selection process

Titles and abstracts were screened against inclusion and exclusion criteria. Full texts were obtained for all that appeared to meet the inclusion criteria or where there was uncertainty. All decisions for excluding reports were recorded. A PRISMA flow diagram (Supplementary Figure 1) presents the data, including information on the number of studies identified, included for data synthesis, reviewed, and excluded (with reasons). LH, PI, and SB were responsible for screening titles and abstracts against inclusion and exclusion criteria. LH, PI, and SB screened one third of titles and abstracts each and screened 10% of each other’s titles and abstracts. LH and PI both screened 50% of all articles at full text review and screened 10% of each other’s full texts to ensure inter-rater reliability. The Kappa statistic was used to determine inter-rater agreement (34). Disagreements were reviewed by SW and LG and resolved through discussion.

#### 2.3.2 Data extraction

Data extraction was conducted using the Joanne Briggs Institute Extraction Form for Prevalence and Incidence Studies. This included study details (lead author and year), methodology (study design, response rate, year of data collection), sample characteristics (mean age, proportion of males), primary outcome measures (alcohol use prevalence, or proportion and 95% confidence intervals, measures used), and secondary outcome measures (burnout, common mental disorders, measures used). If essential data was missing, authors were contacted for further information. LH and PI each completed 50% of the data extraction.

#### 2.3.3 Risk of bias (quality) assessment

The Joanne Briggs Institute critical appraisal checklist for studies reporting prevalence and incidence data was used to determine methodological quality (23). This checklist assessed the following: representativeness of sample, recruitment, adequate sample size, adequate description of subjects and setting, sufficient coverage of sample in data analysis, standard criteria used to measure condition, appropriate statistical analysis, confounding factors, and sub-populations identified using objective criteria. LH and PI each critically appraised 50% of the included studies and checked agreement by critically appraising 10% of the other reviewer’s assessments, resolving any disagreements through discussion. Studies scored between 0 and 59% were considered high risk of bias, 60–79% medium risk of bias, and 80–100% low risk of bias. Studies were not excluded from analyses based on critical appraisal scores.

### 2.4 Data analysis

Separate random-effects meta-analyses were conducted for each outcome to examine the pooled prevalence of (i) hazardous, (ii) harmful, or (iii) dependent alcohol use, and (iv) frequent binge drinking in healthcare professionals. We conducted random-effects (restricted maximum likelihood) meta-analysis using the “metafor” package in R to determine the pooled prevalence of hazardous, harmful, and dependent alcohol use, and frequent binge drinking (based on the measures and cut-offs used by authors, meaning criteria differs across studies). We used the Freeman-Tukey double arcsine transformation on proportions to stabilize variance and ensure extremely large/small proportions had appropriate weighting. Analyses were conducted on transformed data, but backward transformations were conducted for figures and presentation.

Studies were stratified by time-period of data collection, to investigate whether prevalence estimates differed during the COVID-19 pandemic versus prior to the pandemic, if there was sufficient data. In addition, exploratory sub-group analyses were conducted to explore differences in outcomes depending on the occupational groups included in the samples (e.g., doctors, nurses, all healthcare workers), providing the number of studies was sufficient [i.e., minimum of 4 (35)]. Given the variation in the measures used to determine hazardous alcohol use across studies, an exploratory sub-group analysis was conducted to assess differences in pooled prevalence estimates for hazardous alcohol use, depending on whether studies used the AUDIT (either the full 8-item AUDIT or the 3-item AUDIT-C) compared with other measures (e.g., recommended guidelines).

To assess the degree of heterogeneity, the *I*^2^ measure and its CI were used. *I*^2^ ranges from 0 to 100%, with the following cut-offs suggested for low, modest and high heterogeneity: <25% is low, 25–50% is modest, and > 50% is high (36). Significant heterogeneity was determined using *χ*^2^ for *Q*-test, with a conservative significance level (*p* < 0.01) being used due to increased heterogeneity associated with observational studies (37). If the data were sufficient (*N* ≥ 10 for each variable), univariate meta-regressions were conducted to explore whether the prevalence of mental health problems (i.e., depression, anxiety, PTSD) and burnout reported in studies were associated with heterogeneity in outcomes (e.g., higher prevalence of mental health associated with higher prevalence of hazardous alcohol use). In addition, univariate meta-regressions were conducted to explore whether socio-demographic factors (age, gender) and study variables (study quality, response rate) were associated with heterogeneity in outcomes.

Sensitivity analyses were conducted to determine small study biases and influential cases. These included Trim and Fill, Egger’s Regression Test, and examination of influence statistics. Trim and Fill analysis removes (“trims”) any studies which might contribute to funnel plot asymmetry before “filling” any studies to improve symmetry. This provides (i) an estimate of the number of missing studies, and (ii) an adjusted pooled prevalence based on their inclusion. We used the “influence” function in “metafor” to identify any influential effect sizes and removed them to examine their impact on the pooled prevalence estimates. Finally, we conducted Egger’s regression test as a measure of publication bias. Data and analysis scripts are uploaded as Supplementary materials.

## 3 Results

### 3.1 Study characteristics

The initial search identified 9,108 records, after excluding 2,195 duplicates, as displayed in the PRISMA (38) flow diagram (Supplementary Figure 1). After screening against the eligibility criteria, 64 papers were identified as relevant for inclusion, three of which were cohort studies (data were extracted from the most recent wave), and the remainder were cross-sectional studies. The study characteristics are presented in Supplementary Table 1. Some studies included estimates for multiple outcomes (i.e., hazardous alcohol use *and* harmful alcohol use), meaning they were included in each respective meta-analysis. Regarding risk of bias, 47% (*N* = 30) studies were rated as high risk of bias, 48% (*N* = 31) as medium risk of bias, and 5% (*N* = 3) as low risk of bias (Supplementary Table 1).

In total, 14 studies were identified that reported prevalence estimates during the COVID-19 pandemic and 50 studies reported prevalence estimates prior to the COVID-19 pandemic. In addition, 19 studies reported the prevalence of depression, 12 reported the prevalence of anxiety, six reported the prevalence of PTSD, and six reported the prevalence of burnout using the MBI (29) (high emotional exhaustion, high depersonalization, and/or personal accomplishment).

### 3.2 Hazardous drinking

**Figure 1:**
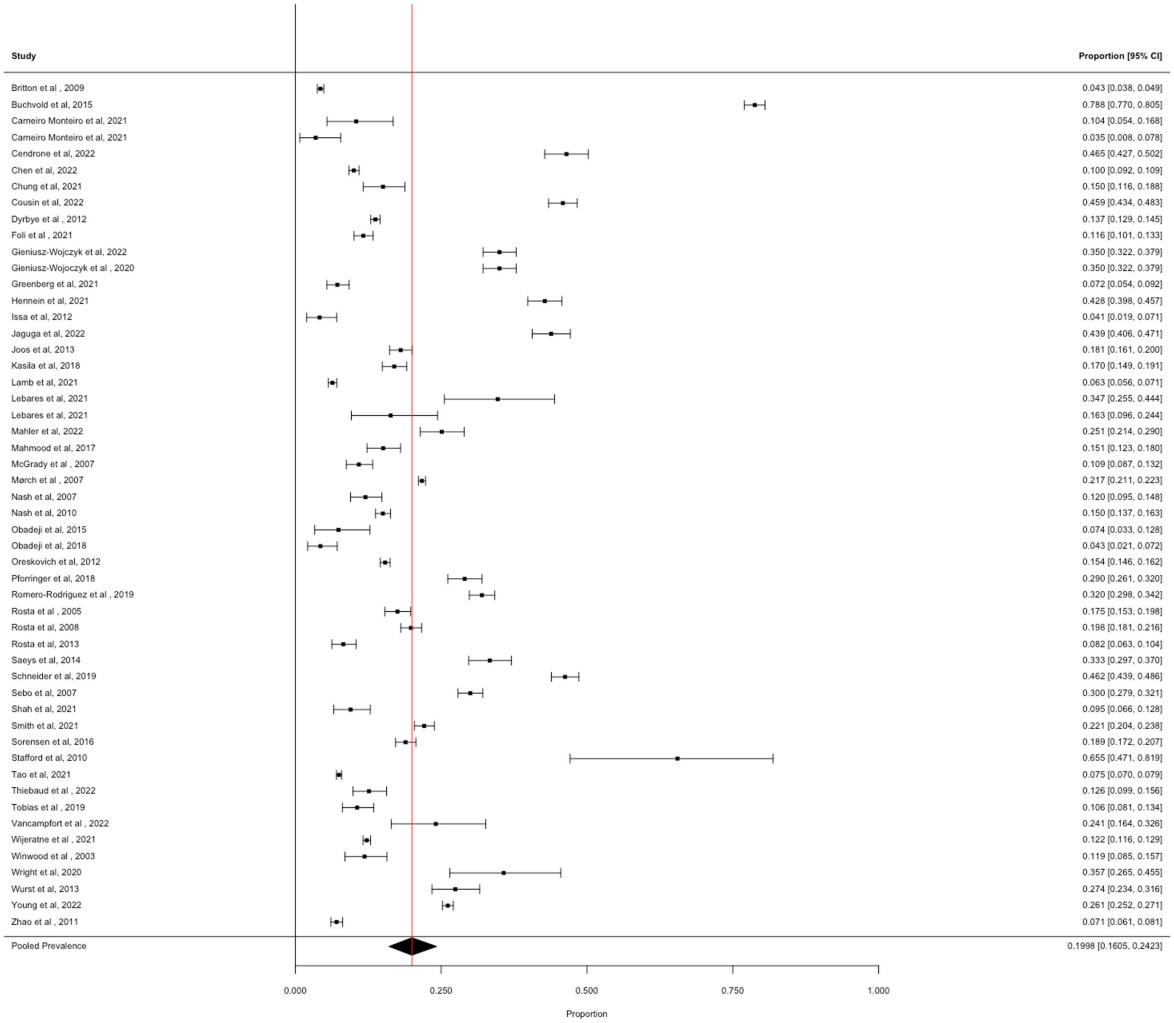
Forest plot showing the prevalence of hazardous alcohol use.

We obtained 52 prevalence estimates for hazardous alcohol use across the identified articles. The pooled prevalence of hazardous alcohol use was 19.98% [95% CI: 16.05 to 24.23%; *I*^2^ = 99.7%], see Figure 1.

#### 3.2.1 Moderator analyses

##### 3.2.1.1 Occupational groups

Comparisons of prevalence estimates across studies of doctors (*N* = 25), nurses (*N* = 7), and all hospital staff (*N* = 10), demonstrated a significant subgroup effect (*X*^2^(2) = 12.18, *p* = 0.002). In studies of doctors, the prevalence estimate was 16.78% (95% CI: 13.41 to 20.43%, *I*^2^ = 99.0%). In studies of nurses, the prevalence estimate was 27.02% (95% CI: 12.98 to 43.93%, I^2^ = 99.8%), and in studies whose samples included all hospital staff, the prevalence estimate was 32.04% (95% CI: 22.57 to 42.32%, *I*^2^ = 99.6%).

##### 3.2.1.2 COVID-19

The comparison of prevalence estimates from studies conducted during the COVID-19 pandemic (*N* = 11) versus studies conducted before the COVID-19 pandemic (*N* = 41) demonstrated a weak subgroup effect (*X*^2^(1) = 3.87, *p* = 0.049), which did not meet our conservative value of p for significance. During the COVID-19 pandemic, the pooled prevalence was 28.19% (95% CI: 19.23 to 38.11%, *I*^2^ = 99.5%), compared with 17.94% (95% CI: 13.82 to 22.47%, *I*^2^ = 99.7%) from before the pandemic.

##### 3.2.1.3 Measures of hazardous drinking

There was no significant difference in prevalence estimates when hazardous alcohol use was determined via the AUDIT vs. other measures, e.g., ASSIST, (*X*^2^(1) 1.56, *p* = 0.210). Pooled prevalence of hazardous alcohol use as measured using the AUDIT (*N* = 44) was 21.10% (95% CI: 16.69 to 25.87%; *I*^2^ = 99.6%), and for other measures (*N* = 8) was 14.43% (95% CI: 7.22 to 23.38%; *I*^2^ = 99.7%).

#### 3.2.2 Sensitivity analyses

##### 3.2.2.1 Measures of Bias and influence

Egger’s regression test was not significant (*Z* = 0.76, *p* = 0.446) and Trim and Fill did not impute any studies. One effect size was identified as influential (Cook’s Distance = 0.243, DFBETA = 0.559). Removal of this effect size slightly reduced the pooled prevalence estimate to 18.96% (95% CI: 15.52 to 22.66%, *I*^2^ = 99.6%).

##### 3.2.2.2 Response rates

There was no significant association between response rates and prevalence of hazardous drinking (N = 42, β < 0.000, 95% CI: −0.002 to 0.003, Z = 0.37, *p* = 0.713).

##### 3.2.2.3 Study quality

There was no significant association between study quality and prevalence of hazardous drinking (*N* = 52, β = 0.002, 95% CI: −0.001 to 0.005, *Z* = 1.12, *p* = 0.261).

##### 3.2.2.4 Demographics

There was no significant association between the mean age of the sample (*N* = 33, *β* < 0.000, 95% CI: −0.011 to 0.001, *Z* = 0.12, *p* = 0.903), or the proportion of males in the sample (*N* = 50, β = −0.001, 95% CI: −0.004 to 0.001, *Z* = 1.58, *p* = 0.114) and prevalence of hazardous drinking.

##### 3.2.2.5 Mental health and burnout

There was no significant association between the prevalence of anxiety and the prevalence of hazardous drinking (*N* = 10, *β* = −0.005, 95% CI: −0.011 to 0.002, *Z* = 1.50, *p* = 0.145). There was no significant association between the prevalence of depression and the prevalence of hazardous drinking (*N* = 13, *β* = 0.002, 95% CI: −0.009 to 0.012, *Z* = 0.31, *p* = 0.756). There were insufficient data to explore the associations between the prevalence of PTSD or burnout with the prevalence of hazardous drinking.

### 3.3 Harmful drinking

**Figure 2:**
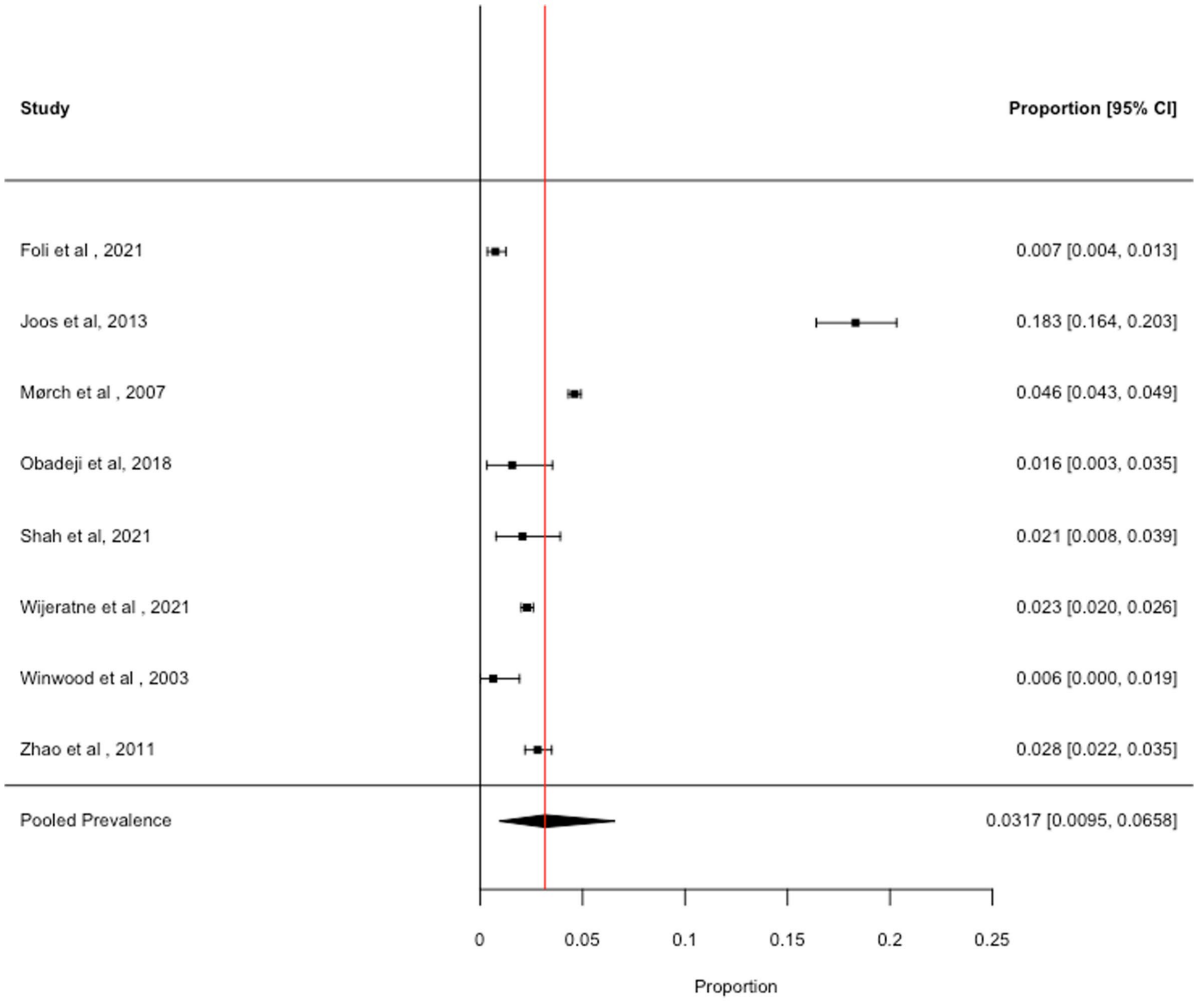
Forest plot showing the prevalence of harmful drinking.

We obtained eight prevalence estimates across the identified articles. The pooled prevalence of harmful alcohol use was 3.17% (95% CI: 0.95 to 6.58%; *I*^2^ = 99.7%), see Figure 2. Removal of one study with high influence scores (Cook’s Distance = 0.755; DFBETA = 2.096) slightly reduced the pooled prevalence estimate (2.03, 95% CI: 1.13 to 3.17%, *I*^2^ = 96.3%). There were insufficient data to conduct sub-group analyses or meta-regressions to explore the impact of the COVID-19 pandemic, burnout, mental health, sociodemographic variables, or variables relating to study quality, on the prevalence of harmful alcohol use.

### 3.4 Dependent drinking

**Figure 3:**
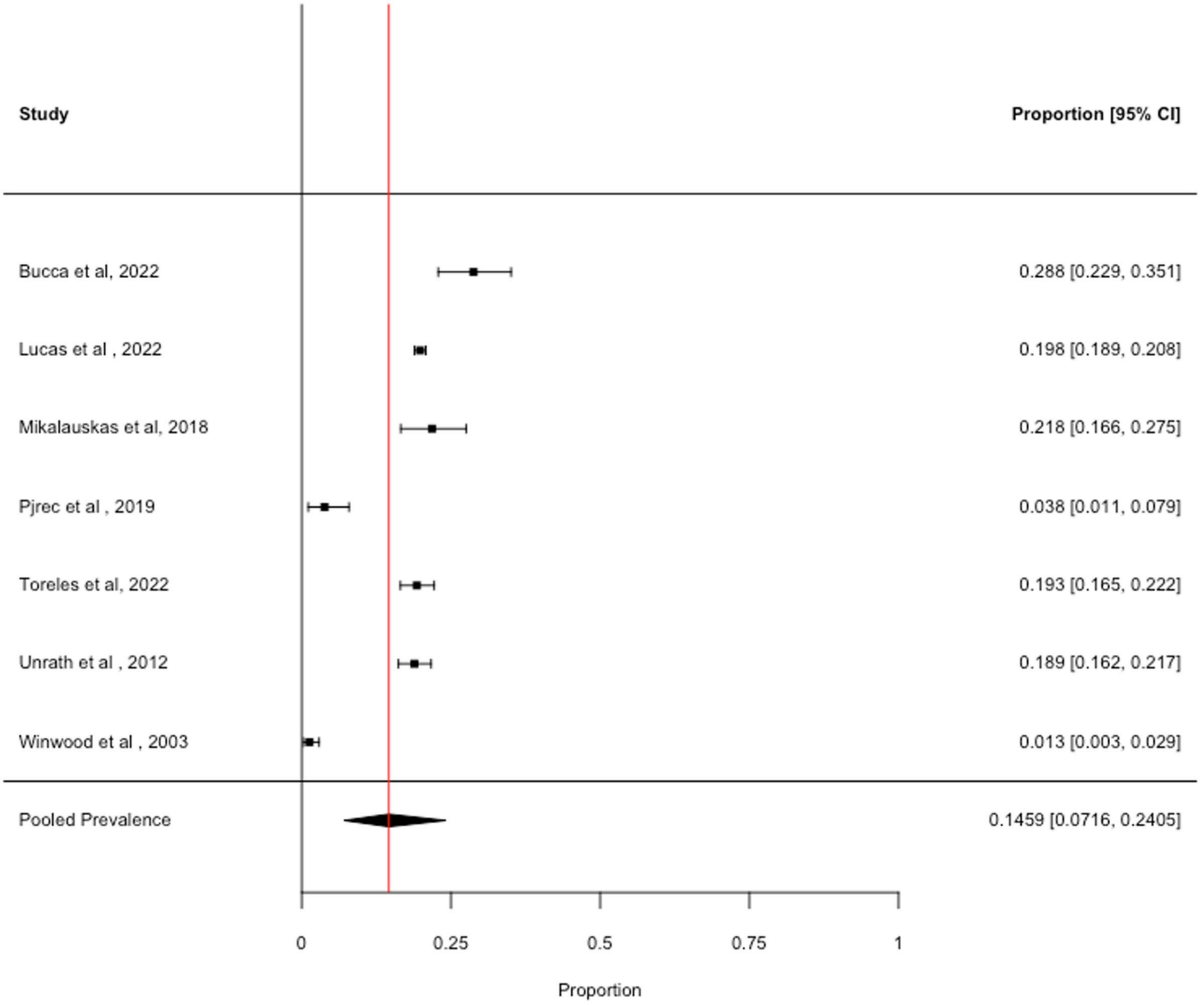
Forest plot showing the prevalence of dependent drinking.

We obtained seven prevalence estimates across the identified articles. The pooled prevalence across dependent alcohol use was 14.59% (95% CI: 7.16 to 25.05%, *I*^2^ = 98.6%), see Figure 3. Removal of one study with high influence scores (Cook’s Distance = 0.587; DFBETA = −1.088) slightly increased the pooled prevalence estimate (18.07, 95% CI: 11.58 to 25.62%, *I*^2^ = 97.2%). We are not confident that this estimate is an accurate indicator of the prevalence of dependent drinking in healthcare professionals, as 5 out of the 7 studies used the CAGE to measure dependent drinking. Guidance suggests that the CAGE is not suitable for use in non-clinical samples (39), which may explain the unreliably high prevalence estimates. It was not possible to examine differences in the prevalence of dependent alcohol use due to the COVID-19 pandemic, burnout, mental health, sociodemographic variables, or variables relating to study quality, due to insufficient data.

### 3.5 Binge drinking

**Figure 4:**
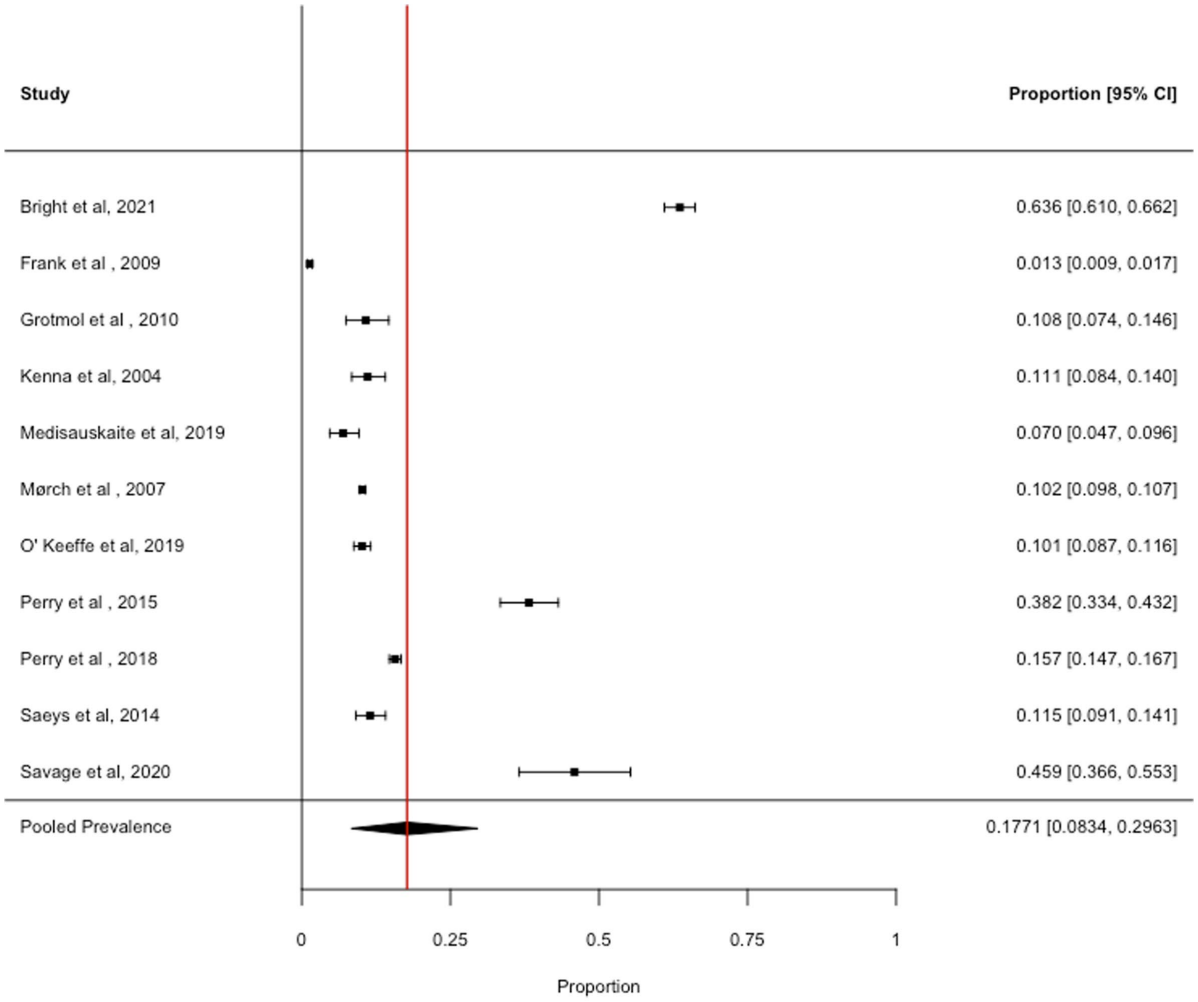
Forest plot showing the prevalence of frequent binge drinking (criteria varied across studies).

We obtained 11 prevalence estimates across the identified articles. The pooled prevalence across binge drinking was 17.71% (95% CI: 8.34 to 29.63%, *I*^2^ = 99.8%), see Figure 4. Removal of one study with high influence scores (Cook’s Distance = 0.486; DFBETA = 0.914) slightly reduced the pooled prevalence estimate (14.04, 95% CI: 7.15 to 22.75%, *I*^2^ = 99.6%). There were insufficient data to address all objectives with binge drinking as the outcome.

## 4 Discussion

### 4.1 Key findings

This international review determined the global prevalence of hazardous, harmful, and dependent alcohol use, and frequent binge drinking within healthcare professionals. A total of 64 studies were eligible for inclusion as they reported at least one prevalence estimate for the outcomes of interest. The pooled prevalence of hazardous alcohol use was 20%, with pooled estimates of 3% for harmful alcohol use, 15% for dependent alcohol use (though these estimates may be unreliable), and 18% for frequent binge drinking. Within studies investigating hazardous, the pooled prevalence of hazardous alcohol use was higher among studies conducted during the COVID-19 pandemic (28%) compared with studies conducted prior to the pandemic (18%). In addition, exploratory analyses showed significant differences in the prevalence of hazardous alcohol use across studies of all healthcare workers (32%) compared with studies of nurses (20%) and doctors (17%). This review examined potential moderators that were hypothesized to be associated with variation in the prevalence of hazardous alcohol use, as this was the only outcome with sufficient data. Response rate, study quality, age (mean), gender (proportion of males), and the prevalence of depression and anxiety were not significantly associated with variance.

Across the world, healthcare professionals have been on the forefront of the COVID-19 pandemic, which has had a detrimental impact on their mental health (19, 20, 40). During previous pandemics/epidemics, healthcare workers reported an increase in health risk behaviors such as drinking alcohol and smoking (17), with adverse psychological consequences lasting for years post-pandemic recovery (21). We now show that the prevalence of hazardous alcohol use among healthcare workers was greater during the COVID-19 pandemic compared with prior to the pandemic. It is critical that healthcare workers are actively monitored, to ensure that those who do suffer with alcohol and/or mental health problems are identified and supported to receive care (3).

Irrespective of the current COVID-19 pandemic, healthcare professionals work under high pressure and intensive conditions, increasing their risk of poor mental health and burnout (3, 4). It was only possible to explore whether the pooled prevalence of depression and pooled prevalence of anxiety were associated with variance in the prevalence of hazardous alcohol use, among healthcare workers, finding no significant effect. However, these analyses were limited as the measures and criteria used to determine the prevalence of depression and anxiety varied across studies, and the exploration of pooled moderation effects may disguise significant associations within individual studies. Within the general population, levels of hazardous drinking are higher in those with a mental health problem, and adults scoring above the ‘probable dependent’ AUDIT cut-off are more than twice as likely to be taking psychotropic medication, and much more likely to be accessing mental health treatment than those scoring below the cut-off (41). Whether levels of co-morbidity differ within healthcare professionals remains relatively unexplored, and an important direction for future research, to ensure that both mental health and alcohol support are available for healthcare professionals and that those needing support are targeted effectively.

We identified significant differences in the prevalence of hazardous alcohol use across different occupational groups, with studies including all healthcare workers obtaining much higher prevalence estimates compared to studies of nurses and studies of doctors. Clinical staff may be less likely than non-clinical staff (e.g., clerical staff, receptionists, caterers, engineers) to disclose their alcohol consumption accurately, through fears of suspension from practice or prejudicing career prospects (9). Additionally, there is some evidence to indicate poorer mental health among non-clinical healthcare professionals during the COVID-19 pandemic (42), meaning this occupational group may be more likely to use alcohol to cope. Somewhat surprisingly, neither age nor gender were significant moderators of prevalence estimates for hazardous alcohol use, contradicting global statistics that have consistently demonstrated that males consume more alcohol than females and are at increased risk of an alcohol dependence (43, 44) and evidenced age-related variation of alcohol use (44). However, the lack of overall moderation effects may result from a lack of variation across all studies to detect differences.

### 4.2 Strengths and limitations

This review followed robust methodological procedures, in line with the Joanna Briggs Institute guidance for systematic reviews of prevalence and incidence data (23), and the PRISMA statement for reporting the findings. In addition, this review was pre-registered with PROSPERO, where the search strategy and statistical analyses were outlined *a priori.* Nevertheless, there were limitations with the review and studies included, which impact the validity of the findings. Due to a lack of financial resources and researcher time, this review was limited to English-only research, which may lead to biased estimates, though only two studies were excluded as English language versions were not available. Given that there were multiple outcomes that resulted in separate meta-analyses, some of which included only a small number of studies, it was not possible to explore all moderators of interest for each outcome. The pooled prevalence estimate for dependent drinking is unreliable, as five out of seven studies used the CAGE to measure dependent drinking, despite guidance stating that it should not be used within non-clinical samples (39). Additionally, there was variation in the criteria used to measure the outcomes, reducing the validity of the pooled prevalence estimates. Furthermore, high levels of heterogeneity were observed, as expected for observational studies (37), despite attempts to explain this through meta-regressions and sub-group analyses. This study found that the prevalence of hazardous drinking was greater in studies conducted during the COVID-19 pandemic, though a large proportion of studies conducted during the pandemic included all healthcare workers, compared with most studies being conducted in doctors and/or nurses before the pandemic, and this sampling imbalance may be a confounder. Response rates varied widely across the included studies, from 6 to 90%, and where response rates were low, the authors rarely used statistical methods to account for or explain low responses. Low response rates among healthcare professionals may reflect confidentiality concerns or fears of disciplinary action (9).

### 4.3 Implications

With the prevalence of hazardous alcohol use being found to be greater during the COVID-19 pandemic compared with prior to the pandemic, findings emphasize the need for workplace interventions aimed at educating healthcare professionals about ‘low-risk’ levels of alcohol use and raising awareness of alcohol-related harms. Such interventions should also focus on adaptive coping strategies, as recent research by Mind demonstrated that 69% of emergency responders felt that their mental health had been negatively impacted by the COVID-19 pandemic, with almost a quarter reporting maladaptive coping strategies, including alcohol use (45). Taken alongside findings from previous pandemics, which indicate that these adverse outcomes could last for years post-pandemic, posing long-term health implications (21), evidence highlights the importance of improving understanding of the relationship between healthcare professionals’ mental health and drinking behaviors, particularly in the context of pandemics, to enable targeted support and recovery.

## 5 Conclusion

This international review identified the pooled prevalence of hazardous, harmful, dependent alcohol use and frequent binge drinking in healthcare professionals across the world, demonstrating that almost one fifth of healthcare professionals drink to hazardous levels and engage in frequent binge drinking. Crucially, the pooled prevalence of hazardous alcohol use was significantly greater among studies conducted during the COVID-19 pandemic compared with pre-pandemic estimates, and further research is needed to investigate whether this is sustained in the post-pandemic period.

## Data availability statement

Data and statistical code are available on Open Science Framework https://osf.io/7ryv8/.

## Author contributions

LH: Conceptualization, Investigation, Methodology, Validation, Visualization, Writing – original draft, Writing – review & editing. PI: Conceptualization, Investigation, Methodology, Project administration, Supervision, Validation, Visualization, Writing – original draft, Writing – review & editing. SB: Conceptualization, Investigation, Methodology, Project administration, Supervision, Validation, Visualization, Writing – review & editing. SW: Conceptualization, Investigation, Supervision, Validation, Visualization, Writing – review & editing. SG: Conceptualization, Investigation, Supervision, Validation, Visualization, Writing – review & editing. LG: Conceptualization, Investigation, Methodology, Project administration, Supervision, Validation, Visualization, Writing – review & editing. AJ: Conceptualization, Formal analysis, Investigation, Methodology, Project administration, Software, Supervision, Validation, Visualization, Writing – original draft, Writing – review & editing.

## Supplementary material

Supplementary Figure 1 Prisma PRISMA 2020 flow diagram (38).

Supplementary Table 1 Study characteristics, separated by outcome measure.

Supplementary Table 2 Search strategy.

Supplementary Table 3 Prisma 2020 for abstracts checklist.
