# Supplementary figures and images for: Hazardous, harmful, and dependent alcohol use in healthcare professionals: a systematic review and meta-analysis
Source: Front Public Health. 2023 Nov 28;11:1304468. doi: 10.3389/fpubh.2023.1304468 (PMC10715281; doi:10.3389/fpubh.2023.1304468)

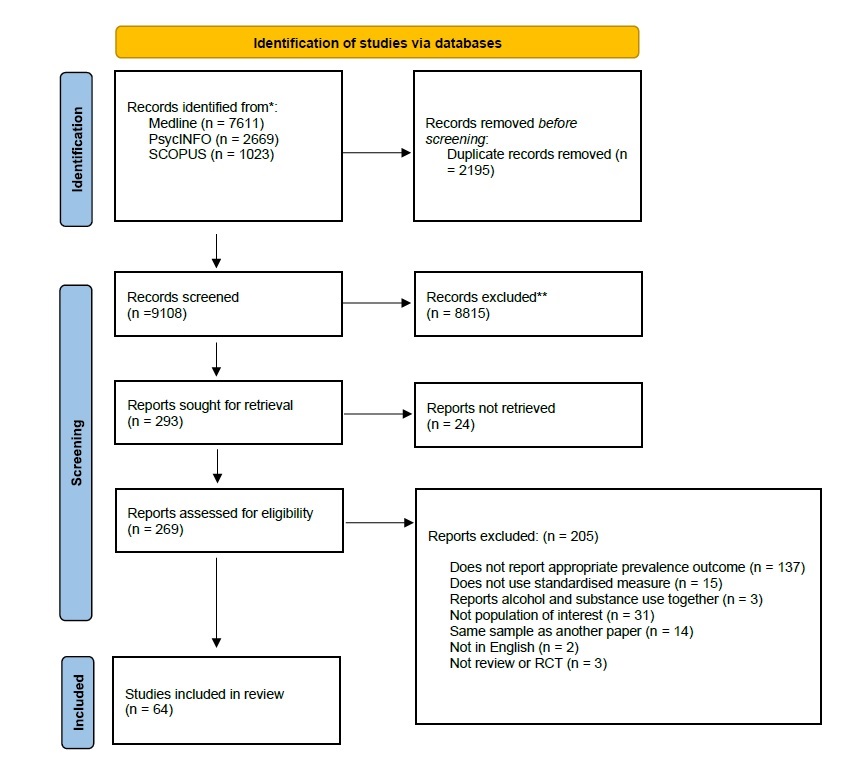

Supplement: Supplementary Figure 1 — Prisma PRISMA 2020 flow diagram (38). [file Image_1.JPEG]
