# Supplementary material for: Hazardous, harmful, and dependent alcohol use in healthcare professionals: a systematic review and meta-analysis
Source: Front Public Health. 2023 Nov 28;11:1304468. doi: 10.3389/fpubh.2023.1304468 (PMC10715281; doi:10.3389/fpubh.2023.1304468)
Supplement: Supplementary Table 1 — Study characteristics, separated by outcome measure. [file Table_1.DOCX]

**Supplementary Table 1.** Study characteristics, separated by outcome measure

| **Author** | **Year published** | **Data collection** | **Country (continent)** | **Study design** | **Occupation** | **Sample size** | **Mean Age** | **Proportion Males %** | **Response Rate %** | **Study quality %** | **Alcohol measure** | **Prevalence** |
| --- | --- | --- | --- | --- | --- | --- | --- | --- | --- | --- | --- | --- |
| ***Hazardous alcohol use*** |  |  |  |  |  |  |  |  |  |  |  |  |
| Gieniusz-Wojczyk et al | 2022 | 2017 | Poland (Europe) | Cross-sectional | Nurses | 1080 | 43 | 3 | 90 | 67 | AUDIT C ≥4 for men, ≥3 for women | 378 (35%) |
| Jaguga et al **COVID-19** | 2022 | 2020 | Kenya (Africa) | Cross-sectional | All hospital staff | 887 | 45 | 45 | NR | 71 | AUDIT ≥8 | 389 (44%) |
| Cendrone et al **COVID-19** | 2022 | 2020 | Italy (Europe) | Cross-sectional | All hospital staff | 682 | 42 | 53 | 79 | 67 | AUDIT C ≥4 for men, ≥3 for women | 317 (46%) |
| Mahler et al **COVID-19** | 2022 | 2020 | Switzerland (Europe) | Cross-sectional | Doctors | 510 | NR | 49 | 51 | 67 | AUDIT C ≥5 for men, ≥4 for women | 128 (25%) |
| Young et al **COVID-19** | 2022 | 2020 | USA (North America) | Cross-sectional | All hospital staff | 8494 | NR | 17 | 28 | 56 | AUDIT C NR | 2220 (26%) |
| Vancampfort et al **COVID-19** | 2022 | 2021 | Uganda (Africa) | Cross-sectional | Nurses | 108 | 35 | 44 | 98 | 78 | AUDIT C ≥4 for men, ≥3 for women | 26 (24%) |
| Thiebaud et al | 2022 | 2018 | France (Europe) | Cross-sectional | Doctors | 515 | 40 | 41 | 51 | 56 | AUDIT ≥8 | 65 (13%) |
| Cousin et al **COVID-19** | 2022 | 2020 | France (Europe) | Cross-sectional | All hospital staff | 1585 | 38 | 21 | 78 | 89 | AUDIT C ≥4 for men, ≥3 for women | 727 (46%) |
| Chen et al | 2022 | 2019 | China (Asia) | Cross-sectional | Psychiatrists | 4520 | 36 | 42 | 65 | 67 | AUDIT C ≥4 for men, ≥3 for women | 453 (10%) |
| Hennein et al **COVID-19** | 2022 | 2020 | USA (North America) | Cross-sectional | All hospital staff | 1092 | 40 | 28 | NR | 67 | AUDIT C ≥4 for men, ≥3 for women | 467 (43%) |
| Lebares et al | 2021 | 2019 | USA (North America) | Cross-sectional | Doctors | 98 | NR | 45 | 65 | 67 | AUDIT C ≥4 for men, ≥3 for women | 34 (35%) |
| Carneiro Monteiro et al | 2021 | 2018 | Brazil (South America) | Cross-sectional | Psychiatrists | 115 | 29 | 49 | 62 | 44 | AUDIT C ≥8 | 4 (3%) |
| Lamb et al **COVID-19** | 2021 | 2020 | UK (Europe) | Cross-sectional | All hospital staff | 4378 | 41 | 25 | 12 | 89 | AUDIT ≥8 | 278 (6%) |
| Shah et al | 2021 | 2020 | Kenya (Africa) | Cross-sectional | Doctors | 338 | 32 | 53 | NR | 78 | ASSIST 11-26 | 32 (9%) |
| Wijeratne et al | 2021 | 2013 | Australia (Oceania) | Cross-sectional | Doctors | 10038 | NR | 52 | 27 | 50 | AUDIT ≥8 | 1228 (12%) |
| Tao et al | 2021 | NR | China (Asia) | Cross-sectional | Doctors | 13980 | 36 | 25 | 67 | 56 | AUDIT C ≥4 for men, ≥3 for women | 1045 (7%) |
| Smith et al **COVID-19** | 2021 | 2020 | USA (North America) | Cross-sectional | All hospital staff | 2246 | 39 | 25 | 10 | 44 | AUDIT C ≥4 for men, ≥3 for women | 496 (22%) |
| Chung et al | 2021 | 2015 | Korea (Asia) | Cross-sectional | Nurses | 386 | NR | 0 | NR | 22 | 14 units per week | 58 (15%) |
| Foli et al | 2021 | NR | United States | Cross-sectional | Nurses | 1478 | 44 | 7 | 34 | 78 | ASSIST 11-26 | 172 (11.6%) |
| Greenberg et al  **COVID-19** | 2021 | 2020 | UK (Europe) | Cross-sectional | Doctors and nurses | 709 | NR | NR | NR | 33 | AUDIT C ≥6 | 51 (7.0%) |
| Wright et al  **COVID-19** | 2020 | 2020 | USA (North America) | Cross-sectional | All hospital staff | 98 | NR | NR | NR | 33 | AUDIT C ≥4 for men, ≥3 for women | 35 (36.1%) |
| Gieniusz-Wojoczyk et al | 2020 | 2017-2018 | Poland (Europe) | Cross-sectional | Nurses | 1080 | 43 | 3 | 90 | 67 | AUDIT C (NR) | 378 (35.0%) |
| Schneider et al | 2019 | 2008-2012 | UK (Europe) | Cross-sectional | Nurses | 1717 | NR | 46 | 55 | 78 | 14/ 21 units per week (women / men) | 794 (46.2%) |
| Tobias et al | 2019 | NR | Brazil (South America) | Cross-sectional | Doctors | 510 | NR | 47 | NR | 67 | AUDIT ≥8 | 54 (10.6%) |
| Romero-Rodriguez et al | 2019 | 2014-2016 | Spain (Europe) | Cross-sectional | Doctors | 1760 | 48 | 37 | 6 | 78 | AUDIT C ≥5 for men, ≥4 for women | 563 (32.0%) |
| Pforringer et al | 2018 | 2016 | Germany (Europe) | Cross-sectional | Doctors | 920 | NR | 55 | 82 | 67 | AUDIT C ≥5 | 267 (29.0%) |
| Kasila et al | 2018 | 2010 | Finland (Europe) | Cross-sectional | All hospital workers | 1233 | 43 | 13 | 54 | 56 | AUDIT C ≥6 for men, ≥5 for women | 209 (17.0%) |
| Obadeji et al | 2018 | NR | Nigeria (Africa) | Cross-sectional | Doctors and nurses | 256 | 35 | 51 | 89 | 67 | AUDIT ≥5 | 11 (4.3%) |
| Mahmood et al | 2017 | 2008-2014 Time(T)4 | Norway (Europe) | Cohort study (follow up: 15 years) | Doctors | 598 at T4 | 43 | 42 | 44 | 75 | AUDIT (9 items) ≥6 for men, ≥5 for women | 90 (15.0%) at T4 |
| Sorensen et al | 2016 | 2014 | Denmark (Europe) | Cross-sectional | Doctors | 1943 | 56 | 48 | 49 | 67 | AUDIT ≥8 | 367 (18.9%) |
| Buchvold et al | 2015 | 2008-2009 | Norway (Europe) | Cross-sectional | Nurses | 2059 | 33 | 9 | 38 | 67 | AUDIT C ≥4 for men, ≥3 for women | 1622 (80.3%) |
| Obadeji et al | 2015 | NR | Nigeria (Africa) | Cross-sectional | Doctors | 122 | 36 | 61 | 90 | 56 | AUDIT ≥5 | 9 (7.3%) |
| Saeys et al | 2014 | 2011 | Belgium (Europe) | Cross-sectional | Doctors | 636 | 45 | 57 | NR | 56 | AUDIT C ≥5 | 212 (34.0%) |
| Joos et al | 2013 | 2011 | Belgium (Europe) | Cross-sectional | Doctors | 1501 | 48 | 53 | 6 | 44 | AUDIT ≥8 for men ≥6 for women | 271 (18.0%) |
| Wurst et al | 2013 | NR | Austria (Europe) | Cross-sectional | Doctors | 456 | 45 | 54 | 18 | 44 | AUDIT ≥8  AUDIT C ≥5 | 61 (13.4%)  125 (27.4%) |
| Rosta et al | 2012 | 2010 | Norway (Europe) | Cohort study (follow up: 10 years) | Doctors | 682 | 53 | 68 | 67 | 67 | AUDIT ≥8 | 56 (8.2%) |
| Issa et al | 2012 | NR | Nigeria (Africa) | Cross-sectional | Doctors | 241 | NR | 76 | 69 | 33 | AUDIT ≥5 | 10 (4.1%) |
| Dyrbye et al | 2012 | 2010 | USA (North America) | Cross-sectional | Doctors | 7179 | NR | 86 | 29 | 71 | AUDIT C ≥5 for men, ≥4 for women | 984 (13.7%) |
| Oreskovich et al | 2012 | 2010 | USA (North America) | Cross-sectional | Doctors | 7197 | NR | 85 | 29 | 38 | AUDIT C ≥5 for men, ≥4 for women | 1108 (15.4%) |
| Zhao et al | 2011 | 2006 | Australia (Oceania) | Cross-sectional | Nurses | 2494 | 43 | 0 | NR | 67 | > Australian guidelines (unreported), risky | 176 (7.1%) |
| Nash et al | 2010 | 2007 | Australia (Oceania) | Cross-sectional | Doctors | 2999 | NR | 70 | 36 | 56 | AUDIT ≥8 | 450 (15.0%) |
| Stafford et al | 2010 | 2008 | Australia (Oceania) | Cross-sectional | Doctors | 29 | NR | 83 | 78 | 56 | AUDIT C ≥5 | 19 (65.5%) |
| Britton et al | 2009 | NR | United States | Cross-sectional | Doctors | 5164 | 58 | 100 | NR | 88 | 2+ drinks per day | 222 (4.3%) |
| Rosta et al | 2008 | 2006 | Germany (Europe) | Cross-sectional | Doctors | 1917 | NR | 61 | 58 | 56 | AUDIT C ≥5 | 380 (19.8%) |
| McGrady et al | 2007 | 2004 | Northern Ireland (Europe) | Cross-sectional | Doctors | 735 | 46 | 65 | 68 | 67 | 14 / 21 units per week (women / men) | 80 (12.6%) |
| Nash et al | 2007 | 2006 | Australia (Oceania) | Cross-sectional | Doctors | 566 | 53 | 65 | 46 | 56 | AUDIT ≥8 | 68 (12.0%) |
| Sebo et al | 2007 | 2002 | Switzerland (Europe) | Cross-sectional | Doctors | 1784 | 51 | 84 | 65 | 78 | AUDIT C ≥5 for men, ≥4 for women | 535 (30.0%) |
| Mørch et al | 2007 | 1993 | Denmark (Europe) | Cross-sectional | Nurses | 17647 | NR | 0 | 86 | 71 | 14 + drinks per week | 3829 (22.0%) |
| Rosta et al | 2005 | 2000 | Norway (Europe) | Cross-sectional | Doctors | 1120 | NR | 69 | 86 | 44 | AUDIT ≥9 | 196 (10.2%) |
| Winwood et al | 2003 | 2001 | Australia (Oceania) | Cross-sectional | Dentists | 312 | 43 | 73 | 64 | 78 | AUDIT ≥8 | 37 (11.0%) |
| ***Harmful alcohol use*** |  |  |  |  |  |  |  |  |  |  |  |  |
| Wijeratne et al | 2021 | 2013 | Australia (Oceania) | Cross-sectional | Doctors | 10038 | NR | 52 | 27 | 50 | AUDIT ≥15 | 230 (2.3%) |
| Shah et al | 2021 | 2020 | Kenya (Africa) | Cross-sectional | Doctors | 338 | 32 | 53 | NR | 78 | ASSIST >27 | 7 (3%) |
| Foli et al | 2021 | NR | United States | Cross-sectional | Doctors | 1478 | 44 | 7 | 34 | 78 | ASSIST >26 | 11 (0.7%) |
| Obadeji et al | 2018 | NR | Nigeria (Africa) | Cross-sectional | Doctors and nurses | 256 | 35 | 51 | 89 | 667 | AUDIT ≥15 | 4 (1.6%) |
| Zhao et al | 2011 | 2006 | Australia (Oceania) | Cross-sectional | Nurses | 2494 | 43 | 0 | NR | 67 | > Australian guidelines (unreported), high-risk | 70 (2.8%) |
| Joos et al | 2013 | 2011 | Belgium (Europe) | Cross-sectional | Doctors | 1501 | 48 | 53 | 6 | 44 | AUDIT ≥15 | 275 (18.3%) |
| Mørch et al | 2007 | 1993 | Denmark (Europe) | Cross-sectional | Nurses | 17647 | NR | 0 | 86 | 71 | 27+ drinks per week | 813 (5.0%) |
| Winwood et al | 2003 | 2001 | Australia (Oceania) | Cross-sectional | Dentists | 312 | 43 | 73 | 64 | 78 | AUDIT 15-19 | 2 (0.6%) |
| ***Dependent alcohol use*** |  |  |  |  |  |  |  |  |  |  |  |  |
| Lucas et al **COVID-19** | 2022 | 2021 | France (Europe) | Cross-sectional | Doctors and nurses | 6935 | 45 | 21 | NR | 56 | CAGE ≥2 | 1375 (20%) |
| Bucca et al **COVID-19** | 2022 | 2021 | USA (North America) | Cross-sectional | All hospital staff | 212 | NR | 30 | 30 | 22 | AUDIT C NR | 61 (29%) |
| Toreles et al **COVID-19** | 2022 | 2021 | Paraguay (South America) | Cross-sectional | Doctors | 747 | 39 | 33 | 83 | 67 | CAGE ≥2 | 144 (19.3%) |
| Pjrek et al | 2019 | NR | Austria (Europe) | Cross-sectional | Doctors | 131 | 50 | 56 | 33 | 56 | CAGE ≥2 | 5 (3.8%) |
| Mikalauskas et al | 2018 | 2017 | Lithuania (Europe) | Cross-sectional | Doctors | 220 | NR | NR | 38 | 44 | CAGE ≥2 | 48 (21.8%) |
| Unrath et al | 2012 | 2009 | Germany (Europe) | Cross-sectional | Doctors | 790 | NR | 70 | 39 | 67 | CAGE-G ≥2 | 149 (18.9%) |
| Winwood et al | 2003 | 2001 | Australia (Oceania) | Cross-sectional | Dentists | 312 | 43 | 73 | 64 | 78 | AUDIT ≥20 | 4 (1.3%) |
| ***Binge drinking*** |  |  |  |  |  |  |  |  |  |  |  |  |
| Bright et al | 2021 | 2019 | UK (Europe) | Cross-sectional | Nurses | 1338 | NR | 12 | 4.5 | 56 | >6 units at least once a month | 851 (63.6%) |
| Savage et al | 2020 | 2018 | UK (Europe) | Cross-sectional | Doctors | 109 | NR | NR | 24 | 11 | >6 units (women) or >8 units (men) at least once in previous month | 50 (46.0%) |
| Medisauskaite et al | 2019 | 2016 | UK (Europe) | Cross-sectional | Doctors | 417 | 47 | 48 | NR | 33 | 6+ drinks per occasion (no time frame) | 29 (35.4%) |
| O' Keeffe et al | 2019 | NR | Ireland (Europe) | Cross-sectional | Doctors | 1749 | NR | 51 | 55 | 66 | 6+ drinks on once a week / multiple times a week | 177 (10.1%) / 58 (3.3%) |
| Perry et al | 2018 | 2014-2015 | Australia (Oceania) | Cross-sectional | Nurses | 5041 | 48 | 91 | NR | 63 | 5+ drinks on one occasion in previous month | 791 (15.7%) |
| Perry et al | 2015 | NR | Australia (Oceania) | Cross-sect5ional | Nurses | 382 | 40 | 17 | 31 | 33 | 5+ drinks on one occasion in previous month | 146 (39.2%) |
| Saeys et al | 2014 | 2011 | Belgium (Europe) | Cross-sectional | Doctors | 636 | 45 | 57 | NR | 56 | ≥5 drinks every week / every month | 17 (3.0%) / 73 (12.0%) |
| Grotmol et al | 2010 | NR | Norway (Europe) | Cohort study (follow up: 9.5 years) | Doctors | 288 | 31 | 43 | 79 | 50 | ≥5 units in one session, 2 + times per month. | 31 (8.0%) |
| Frank et al | 2009 | 2007-2008 | Canada (North America) | Cross-sectional | Doctors | 3123 | NR | 66 | 41 | 78 | 5+ drinks on typical drinking occasion | 41 (1.3%) |
| Mørch et al | 2007 | 1993 | Denmark (Europe) | Cross-sectional | Doctors | 17647 | NR | 0 | 86 | 71 | 4+ drinks on last weekday / 10 + drinks on last weekend day | 1536 (10.0%) / 1800 (13.0%) |
| Kenna et al | 2004 | 2002 | United States | Cross-sectional | All hospital staff | 479 | NR | NR | 69 | 67 | 5+ drinks at least once in past month | 53 (11.1%) |

NR; Not Reported. UK; United Kingdom. USA; United States of America. AUDIT; Alcohol Use Disorder Identification Test. AUDIT-C; Alcohol Use Disorder Identification Test-Consumption. ASSIST; Alcohol, Smoking, and Substance Involvement Screening Tool. CAGE; Cut, Annoyed, Guilty, Eye Questionnaire.
