# Supplementary material for: Hazardous, harmful, and dependent alcohol use in healthcare professionals: a systematic review and meta-analysis
Source: Front Public Health. 2023 Nov 28;11:1304468. doi: 10.3389/fpubh.2023.1304468 (PMC10715281; doi:10.3389/fpubh.2023.1304468)
Supplement: Supplementary Table 2 — Search strategy. [file Table_2.DOCX]

**Supplementary materials**

**Search Strategy**

**Medline:** TITLE-ABS-KEY (health adj3 worker* OR health adj3 professional* OR doctor* OR nurse* OR physician* OR “general practitioner*” OR surgeon* OR “medical professional*” OR “dentist*” or “pharmacist”) AND (alcohol* OR “harmful drink*” OR “alcohol dependent*” OR "hazardous drink*" OR "problem* drink*" OR "risk* drink*" OR "binge* drink*") NOT (intervention OR student*)

**Records identified:** 7611

**PsycINFO:** TITLE-ABS-KEY (health adj3 worker* OR health adj3 professional* OR doctor* OR nurse* OR physician* OR “general practitioner*” OR surgeon* OR “medical professional*” OR “dentist*” or “pharmacist”) AND (alcohol* OR “harmful drink*” OR “alcohol dependent*” OR "hazardous drink*" OR "problem* drink*" OR "risk* drink*" OR "binge* drink*") NOT (intervention OR student*)

**Records identified:** 2669

**Scopus:** TITLE-ABS-KEY (health w/3 worker* OR health w/3 professional* OR doctor* OR nurse* OR physician* OR “general practitioner*” OR surgeon* OR “medical professional*” OR “dentist*” or “pharmacist”) AND (alcohol* OR “harmful drink*” OR “alcohol dependent*” OR "hazardous drink*" OR "problem* drink*" OR "risk* drink*" OR "binge* drink*") NOT (intervention OR student*)

**Records identified:** 1023
